# Vanishing Twin Syndrome After In Vitro Fertilization and Embryo Transfer: Mechanisms, Perinatal Consequences, Diagnostic Pitfalls, and Prevention

**DOI:** 10.3390/biomedicines14092036

**Published:** 2026-09-10

**Authors:** Kristóf Bereczki, Mátyás Bukva, Krisztina Reuter, Csaba Bereczki, Bálint Kolcsár

**Affiliations:** 1 Department of Obstetrics and Gynecology, Albert Szent-Györgyi Medical School, University of Szeged, H-6725 Szeged, Hungarykolcsar.balint@gmail.com (B.K.); 2 Department of Immunology, Albert Szent-Györgyi Medical School, Faculty of Science and Informatics, University of Szeged, H-6720 Szeged, Hungary; bukvamatyas@gmail.com; 3 Department of Pediatrics and Pediatric Health Center, University of Szeged Albert Szent-Györgyi Health Center, H-6725 Szeged, Hungary; 4 Laboratory of Microscopic Image Analysis and Machine Learning, Institute of Biochemistry, Biological Research Centre, Hungarian Research Network (HUN-REN), H-6726 Szeged, Hungary

**Keywords:** vanishing twin syndrome, in vitro fertilization (IVF), embryo transfer, embryo development, placental development, fetal growth restriction, pregnancy complications

## Abstract

Vanishing twin syndrome (VTS) denotes the spontaneous loss of one conceptus from an initially recognized multiple pregnancy, most often during the first trimester. Its clinical relevance has increased with in vitro fertilization and embryo transfer (IVF-ET), as multiple embryo transfer creates the substrate for dizygotic VTS, while blastocyst transfer may be followed by monozygotic splitting and serial early ultrasonography increases detection. However, the literature remains heterogeneous, with empty gestational sacs, losses after fetal cardiac activity, and later single fetal demise often grouped together despite different biological and prognostic implications. New evidence published in recent years on frozen embryo transfer outcomes, cell-free DNA screening, and early childhood development warrants an updated synthesis of vanishing twin syndrome to inform individualized risk assessment, counselling, and standardized research reporting. Overall, the surviving singleton appears to have a lower risk than an ongoing twin pregnancy but a higher risk than a primary singleton, particularly for preterm birth, low birthweight, and small-for-gestational-age birth, with risk increasing when loss occurs after fetal cardiac activity or later in gestation. Nevertheless, evidence is not unanimous, as several well-characterized cohorts and earlier meta-analyses reported no measurable perinatal disadvantage. Two reports from a single tertiary center, based on substantially overlapping recruitment periods, further suggest that VTS may be proportionally less frequent among established twin pregnancies after IVF/ICSI than after spontaneous conception, while IVF-associated VTS has been linked to placental abnormalities, diabetes, and fetal growth restriction; this observation requires independent confirmation. Contemporary frozen embryo transfer cohorts indicate that VTS remains relevant in modern practice, whereas limited long-term data have not demonstrated impaired early childhood growth or development. Management should document the initial number of gestational sacs, embryonic structures, cardiac activity, chorionicity, and timing of loss, while adapting aneuploidy screening to residual trophoblastic DNA and individualizing fetal-growth surveillance. Elective single-embryo transfer remains the most effective preventive strategy, although it cannot eliminate monozygotic twinning. Standardized definitions and long-term offspring follow-up are needed.

## 1. Introduction

Vanishing twin syndrome (VTS) describes the spontaneous disappearance of one or more conceptuses after ultrasonographic recognition of a multiple pregnancy, with continuation of the pregnancy as a singleton or a lower-order multiple gestation [1,2]. The phenomenon was first quantified when early sonography became routine: of twin gestations identified before 10 weeks, 71% were delivered as singletons [3]. The label is clinically convenient but biologically imprecise. A second empty sac at 6 weeks, resorption of an embryo after documented cardiac activity at 10 weeks, and single fetal demise near the end of the first trimester do not represent equivalent exposures. Nevertheless, all have been classified as VTS in published cohorts, producing large differences in reported incidence and outcome.

**Figure 1:**
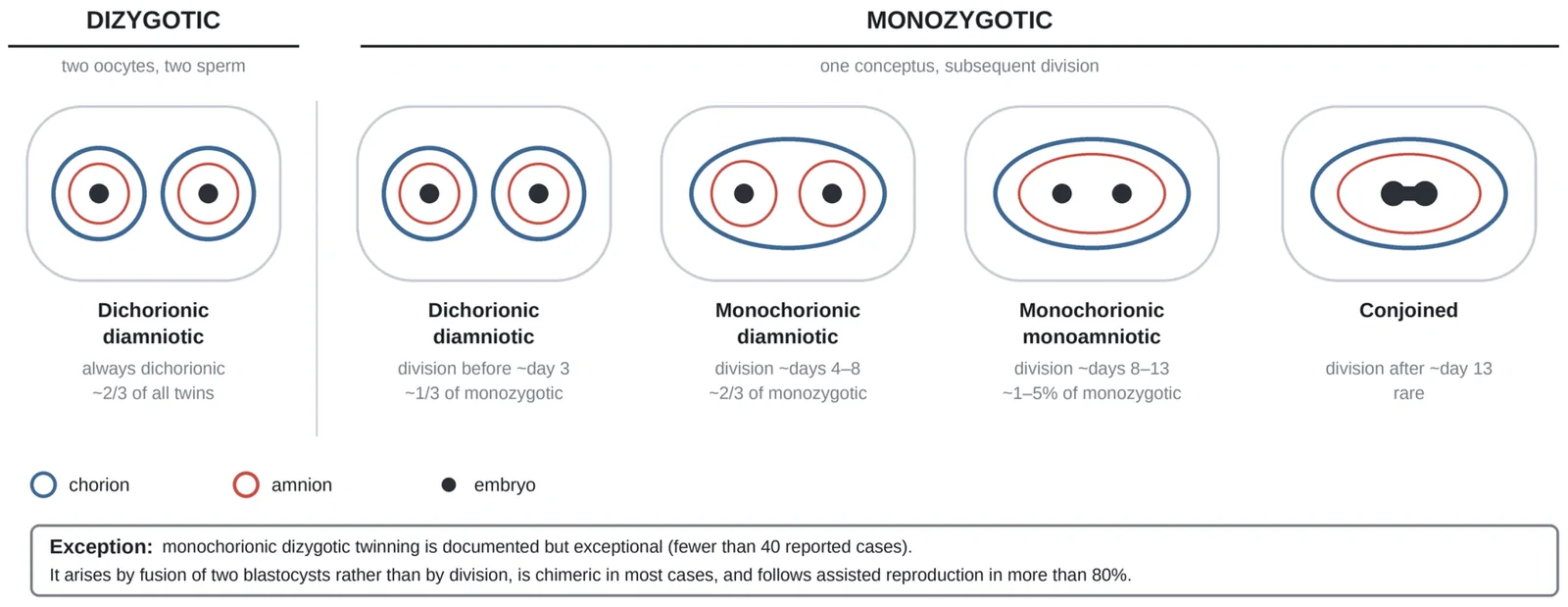
Types of twinning, their membrane arrangements, and the mechanisms by which they arise. Dizygotic twins follow fertilization of two oocytes and are conventionally dichorionic and diamniotic. Monozygotic twins arise from a single conceptus, and chorionicity and amnionicity have traditionally been attributed to the timing of division, although this model is contested. Monochorionic dizygotic twinning is exceptional, arises by fusion of two blastocysts rather than by division, and occurs predominantly after assisted reproduction.

Because VTS arises from a multiple gestation, its interpretation depends on how that gestation was formed (Figure 1). Dizygotic twins follow fertilization of two oocytes and are conventionally always dichorionic and diamniotic. Monozygotic twins arise from a single conceptus, and their membrane arrangement has traditionally been ascribed to the timing of division: before approximately day 3 producing dichorionic diamniotic twins, between approximately days 4 and 8 monochorionic diamniotic twins, between approximately days 8 and 13 monochorionic monoamniotic twins, and later still conjoined twins [4]. This timing model is no longer uncontested. Fusion-based and fertilization-based alternatives have been proposed, and dichorionic monozygotic pregnancies after single blastocyst transfer are difficult to reconcile with early division alone, so the day ranges are best presented as convention rather than as established mechanism [4,5]. Monozygotic twinning occurs in approximately 4 per 1000 deliveries worldwide and varies comparatively little between populations, whereas dizygotic twinning accounts for most geographic and temporal variation and is increased by maternal age, elevated endogenous follicle-stimulating hormone, and ovulation induction [6].

Monochorionic dizygotic twinning, which is occasionally listed as a separate category, is exceptional rather than routine: fewer than 40 cases have been reported, it is attributed to fusion of two blastocysts rather than to any division event, chimerism is demonstrable in most, and more than 80% follow assisted reproduction [7]. Two practical consequences follow for VTS research. Sex discordance does not exclude monochorionicity, and chorionicity should therefore be assigned sonographically before 14 weeks rather than inferred from zygosity [8]. In addition, because shared placental circulations make the loss of one conceptus a qualitatively different event, chorionicity is a prerequisite for interpreting any VTS cohort rather than an optional descriptor.

The issue is particularly relevant to in vitro fertilization and embryo transfer (IVF-ET). Assisted reproduction increases the absolute number of recognized early multiple implantations through the transfer of more than one embryo and through intensive early ultrasonographic follow-up [9]. At the same time, infertility, maternal age, embryo competence, ovarian stimulation, endometrial preparation, and placentation may independently influence outcome. Assisted reproduction has itself been associated with altered trophoblast invasion and impaired uterine spiral-artery remodeling, a placental phenotype invoked to explain the excess of pre-eclampsia, fetal growth restriction, and abnormal placentation reported in ART pregnancies, although the relative contributions of the treatment and of the underlying infertility remain unresolved [10]. The appropriate comparator is therefore critical: a surviving singleton after VTS may be compared with a primary singleton, an ongoing twin pregnancy, or VTS after spontaneous conception, and each comparison answers a different clinical question [1,11,12]. This review synthesizes peer-reviewed evidence with emphasis on IVF-ET, critically integrates the studies that compared VTS after IVF/ICSI with VTS after spontaneous conception, and translates the evidence into prevention, prenatal screening, and surveillance.

## 2. Scope and Literature Approach

A focused narrative search of PubMed/MEDLINE, Embase, Web of Science, and the Cochrane Library was conducted through 25 July 2026 using combinations of “vanishing twin”, “spontaneous reduction”, “IVF”, “ICSI”, “embryo transfer”, “fresh transfer”, “frozen embryo transfer”, “preterm birth”, “growth restriction”, and “prenatal screening”, without date restriction and limited to English-language records. Reference lists of major reviews and meta-analyses were examined for earlier landmark cohorts, and forward citation tracking was applied to the pivotal studies. Only peer-reviewed journal publications were used: original studies, systematic reviews, meta-analyses, and society recommendations. Priority was given to studies with an ART-conceived comparator, explicit timing of loss, large samples, or multivariable adjustment; where cohorts overlapped, the largest or most recent report was preferred. Screening and selection were performed by the authors by consensus. A complementary Scite search identified 266 article records mentioning “vanishing twin” or “vanishing twins” in the title or abstract across all publication years; this figure was used to characterize the breadth of the literature search rather than as a formal screening count, as Scite records my include conference abstract, commentaries, and duplicate entries. These search-yield figure is reported to indicate the selectivity of screening and not as a systematic-review flow diagram. Because definitions, ultrasound schedules, and outcomes were not sufficiently uniform for valid de novo pooling, the present work is a critical narrative review rather than a systematic review, and no formal risk-of-bias scoring or quantitative synthesis was undertaken.

## 3. Definition, Diagnosis, and Epidemiology

VTS is diagnosed longitudinally: an early scan demonstrates at least two gestational sacs or embryos, whereas a subsequent scan demonstrates cessation of development, collapse, or disappearance of one conceptus [1,2]. A single late scan cannot reconstruct the initial implantation number. Studies should distinguish an empty sac from a sac containing a yolk sac, fetal pole, or embryo with cardiac activity; record the gestational age at demise; and separate spontaneous loss from selective or multifetal pregnancy reduction, for which the international glossary provides agreed terminology [13]. A pragmatic and already validated dichotomy is available from prenatal screening practice, in which a vanishing twin is classified as type I when an additional empty gestational sac is seen and as type II when an additional non-viable embryo is identified [14]; adopting this nomenclature across obstetric and reproductive studies would remove much of the present heterogeneity. Chorionicity should also be reported, because shared placental vascular connections make demise in a monochorionic pregnancy more consequential than resorption of one dichorionic implantation: after single intrauterine death in monochorionic twins, pooled data from 23 studies and 1294 pregnancies show substantial risks of preterm birth, abnormal fetal brain imaging, and adverse neurodevelopment in the survivor, although all co-twins with normal prenatal imaging had normal neurodevelopment [15]. A proposed minimum reporting set is summarized in Table 1.

Reported prevalence is commonly 15–35% among clinically recognized twin pregnancies, but estimates vary with the timing of the first scan, the diagnostic threshold, and the denominator [1]. Serial scans beginning soon after a positive pregnancy test detect transient sacs that routine obstetric care misses. IVF-ET therefore increases ascertainment even when the conditional probability of loss among established twin pregnancies is not higher. In 78 IVF/intracytoplasmic sperm injection (ICSI) VTS pregnancies and 228 spontaneously conceived VTS pregnancies accumulated over 22 years, Márton et al. reported that VTS represented 12.6% of IVF/ICSI twin pregnancies versus 18.2% of spontaneous twin pregnancies [12]. Their expanded cohort reported the same direction—14.5% versus 22.0%, respectively [16]. These observations challenge the shorthand claim that IVF itself necessarily increases the proportion of twin gestations that undergo spontaneous reduction, although both estimates derive from a single center and neither was standardized to a common first-scan interval.

It is reasonable to ask whether spontaneous reduction is simply background embryonic attrition acting on two conceptuses rather than a distinct phenomenon. A direct comparison with published miscarriage rates is precluded by definition, because the international glossary counts the loss of two embryos in a twin pregnancy as a single miscarriage and states explicitly that the intrauterine loss of one embryo from a multiple gestation is not a miscarriage [13]; registry miscarriage rates therefore exclude vanishing twins from the numerator by construction. Re-expressed per conceptus, however, the comparison becomes informative and supports the interpretation that VTS largely reflects ordinary early attrition. In 1597 IVF/ICSI pregnancies, loss per gestational sac in twin pregnancies was 11.1%, falling to 7.3% once fetal cardiac activity had been documented, against 21.7% of singleton pregnancies that failed to progress [17]; an independent cohort of 1593 pregnancies after day-3 double-embryo transfer reported loss of 11.46% per sac in twins versus 18.5% in singletons [18]. Both estimates lie within the range of clinical miscarriage reported in large unselected populations, of the order of 10–13% between the ages of 25 and 34 [19].

Two qualifications keep this framing honest. First, if each conceptus were lost independently with probability p, the observed proportions of twin pregnancies reducing to singletons would imply p of roughly 6–10%, which is compatible with background attrition; yet the observed frequency of complete twin loss in the same cohorts exceeds the square of that probability by severalfold, indicating that the two conceptuses share maternal, uterine, and endocrine failure modes and are not independent [17,18]. Second, reported VTS frequencies are dominated by ascertainment. Estimates of the proportion of ART singletons originating from a twin gestation range from approximately 3% to 10% across cohorts using the same denominator, differing chiefly in how early and how systematically the first scan was performed [20,21]. Cross-study comparisons of VTS frequency therefore measure surveillance intensity at least as much as biology, which is a further argument for the standardized first-scan reporting proposed in Section 11.

## 4. Why IVF-ET Changes the Biological and Clinical Context

IVF-ET can generate VTS by two routes. After double- or multiple-embryo transfer, more than one embryo may implant but only one continues. After single-embryo transfer, a transferred embryo may split, creating a monozygotic twin gestation followed by loss of one twin. In a national cohort of 28,596 fresh single-embryo-transfer pregnancies, monozygotic twinning was more frequent after day 5–6 than day 2–3 transfer (2.50% versus 1.71%), and assisted hatching was associated with increased risk in cleavage-stage transfers [22]. A meta-analysis of 40 studies confirmed blastocyst-stage transfer, younger female age, conventional IVF rather than ICSI, and assisted hatching as risk factors for monozygotic twinning [23], and the largest contemporary registry analysis—154,671 live births after single embryo transfer—found a monozygotic twinning rate of 1.5%, with blastocyst transfer and fresh (rather than vitrified-warmed) transfer as the principal determinants [24]. Elective single-embryo transfer therefore markedly reduces, but cannot abolish, the biological substrate for VTS.

Several mechanisms may coexist. Embryonic aneuploidy is a plausible proximate cause of early loss, consistent with the relation between maternal age and chromosomal abnormalities in IVF pregnancy loss, in which 71% of karyotyped losses after documented cardiac activity were chromosomally abnormal [25]. Embryo morphology also reflects developmental competence: transfer of top-quality rather than intermediate-quality embryos was associated with lower early loss rates among twin pregnancies, although overall loss rates did not differ by embryo grade and the effect was confined to specific strata [26]. These observations offer partial support for the interpretation that laboratory selection of apparently viable embryos may contribute to the lower conditional VTS prevalence in IVF/ICSI twins [12]. Morphology, however, is not a chromosomal diagnosis, and even euploid embryos can fail through mosaicism, epigenetic disturbance, abnormal placentation, or nonembryonic maternal factors.

Preimplantation genetic testing for aneuploidy (PGT-A) has become common in some programs and should change the composition, if not necessarily the frequency, of early loss. Because roughly half to two-thirds of first-trimester losses are chromosomally abnormal [27], transferring embryos screened as euploid should remove a substantial share of the aneuploid conceptuses that would otherwise implant and then arrest, shifting the residual causes of VTS toward non-chromosomal mechanisms such as abnormal placentation, hemorrhage, or maternal factors. It does not follow that PGT-A reduces the incidence of VTS. Reductions in miscarriage after PGT-A are in large part a denominator effect: in a randomized trial in women aged 36 to 40, fewer women reached transfer and fewer miscarried, while live birth was identical at 24% in both arms [28], and in a randomized trial of 1212 women with a good prognosis, cumulative live birth was 77.2% with PGT-A versus 81.8% without [29]. Two mechanisms also act in opposite directions on VTS specifically: euploid single-embryo transfer reduces dizygotic multiple implantation, whereas PGT-A obliges blastocyst-stage transfer, which is a replicated risk factor for monozygotic twinning, even though embryo biopsy itself has not been shown to increase it [23,24]. We identified no study reporting VTS or spontaneous reduction rates in PGT-A cycles; the directly relevant literature is confined to a case report of monozygotic VTS after single euploid blastocyst transfer [30], and PGT-A outcome cohorts frequently exclude vanishing-twin pregnancies by design. Reporting PGT-A status, as proposed in Table 1, is therefore a prerequisite for interpreting contemporary VTS incidence.

A second pathway is placental and hemostatic. Multiple implantation sites may compete for endometrial space and perfusion, and demise may leave hemorrhage, inflammation, altered vascular remodeling, or persistent trophoblast. Clinical data support at least the hemorrhagic component: in 1078 IVF twin pregnancies matched 1:1 for the presence of first-trimester intrauterine hematoma and drawn from 4128 screened pregnancies, hematoma was associated with a higher incidence of VTS (145/539, 26.9%, versus 101/539, 18.7%), and within the resulting VTS subgroup the surviving singleton was more often delivered preterm (57/145, 39.3%, versus 20/101, 19.8%) [31]; subchorionic hematoma in IVF twin pregnancies has likewise been linked to early twin pregnancy loss [32]. The surviving fetus may therefore inherit a placenta formed in a multiple-implantation environment even after the second sac disappears. This model is consistent with the placental phenotype described after fresh IVF, in which VTS pregnancies showed more small placentas and more anatomic placental abnormalities than IVF singletons (adjusted odds ratio 2.15, 95% CI 1.08–4.28) [33], and with the broader excess of anatomic and vascular placental pathology observed in ART pregnancies generally [34]. It also suggests why timing may matter, because loss after substantial placental development is a larger physiological event than arrest before a fetal pole or heartbeat is visible. This last inference should nevertheless be stated cautiously: in the same fresh-IVF cohort, VTS was not associated with preterm birth or growth restriction, and loss after 8 weeks was not associated with greater placental pathology [33]. Mechanistic accounts of endometrial competition and persistent trophoblast remain hypothetical and have not been tested directly in human ART pregnancies.

Specifying what would falsify these models is more useful than restating that they are unproven. For endometrial competition, the informative measurements have not been made: no human study has related inter-sac distance at 6 to 7 weeks, or sac-specific subendometrial perfusion measured by three-dimensional power Doppler or contrast-enhanced ultrasound, to which conceptus subsequently vanishes, even though intervillous contrast imaging is feasible from 6 weeks and regional mapping of the uterus before transfer has been demonstrated. The single reported signal, an association between endometrial thickness below 10 mm and spontaneous reduction, derives from 69 pregnancies and has not been replicated. Endometrial receptivity transcriptomic testing cannot fill this gap, since it has not improved live birth in randomized trials and has never been evaluated against the number of implantations rather than a binary pregnancy outcome [35]. For persistent trophoblast, the strongest available evidence is indirect but consistent: after selective reduction in dichorionic twins, the cell-free DNA fraction attributable to the demised fetus peaks 7 to 9 weeks after demise and remains detectable for as long as 16 weeks [36], which is irreconcilable with the minutes-long half-life of circulating placental DNA and therefore implies continued release from surviving trophoblast. Cell-free DNA has nonetheless never been used deliberately to date a demise or to quantify residual trophoblast mass. Angiogenic markers are a further gap: to our knowledge, soluble fms-like tyrosine kinase-1, placental growth factor, and their ratio have not been reported in vanishing-twin pregnancies, despite validated assays and published twin reference ranges. Finally, no placental pathology study has applied the Amsterdam Placental Workshop Group criteria to a documented first-trimester vanishing twin [37], and the existing VTS placental cohort used an institutional nosology and reported no lesion-level prevalences [33]. Tractable endpoints therefore exist: inter-sac distance and per-sac perfusion at 6 to 7 weeks; serial demised-twin cell-free DNA fraction to undetectability; gestational-age-specific angiogenic ratios in VTS, ongoing twins, and singletons; and Amsterdam-classified placental findings with cord-insertion distance in VTS placentas against matched ART singletons.

## 5. Obstetric and Perinatal Outcomes

### 5.1. Cohort Evidence and the Influence of Study Design

The most reproducible pattern is a risk gradient. Compared with ongoing twins, VTS survivors usually have longer gestation and higher birthweight; compared with primary singletons, they may have higher risks of preterm birth, low birthweight, and small-for-gestational-age birth. In early Danish IVF/ICSI data, VTS was associated with low and very low birthweight, especially when loss occurred after 8 weeks, and the adjusted odds of death during follow-up were 3.6 (95% CI 1.7–7.6) in survivors compared with primary singletons, with a non-significant increase in cerebral palsy (odds ratio 1.9, 95% CI 0.7–5.2) [20]. A subsequent national analysis identified VTS as a predictor of small-for-gestational-age birth among IVF singletons (odds ratio 1.50, 95% CI 1.03–2.20), with risk increasing with gestational age at the time of vanishing [38]. In a general obstetric population comprising 278 VTS pregnancies, 1801 dichorionic twin pregnancies, and 252,994 singleton deliveries, in which fertility treatment and maternal age were controlled for, VTS remained an independent risk factor for gestational diabetes, intrauterine growth restriction, very low birthweight, low Apgar score, and perinatal mortality [39]. By contrast, a selected IVF cohort restricted to deliveries at or beyond 24 weeks found perinatal and peripartum outcomes similar to those of primary singletons and clearly better than those of ongoing twins [40]. This inconsistency is expected when studies use different entry scans, exclude early losses, restrict analysis to live births, or combine empty sacs with demise after fetal cardiac activity.

### 5.2. Pooled Estimates and the Role of Timing

Meta-analyses help define the average association but also expose its heterogeneity. The first pooled analysis of ART-conceived pregnancies, based on only five studies, found no significant difference in gestational age or in the overall preterm delivery rate between VTS survivors and singletons-at-start; extremely preterm delivery was more frequent (risk ratio 3.5, 95% CI 1.72–7.12) and mean birthweight marginally lower, but the authors emphasized that these effects were unstable on sensitivity analysis and possibly affected by publication bias [41]. In a larger synthesis of 17 observational studies comprising more than 60,000 ART singletons, the effect of VTS depended on the timing of loss and on the intrauterine growth stage reached: when loss occurred at or before 14 weeks there was no gestational-age penalty, whereas later loss was associated with shorter gestation and greater risks of preterm birth and low birthweight [42]. Importantly, an empty sac was not equivalent to loss of an embryo with documented cardiac activity. A third ART meta-analysis of 15 studies estimated higher odds of preterm birth before 37 weeks (odds ratio 2.41, 95% CI 1.46–3.99) and of small-for-gestational-age birth (odds ratio 2.70, 95% CI 1.39–5.24) relative to ART singletons, while the pooled estimate for congenital malformations did not show a clear increase (odds ratio 1.25, 95% CI 0.87–1.79) [43]. Taken together, these results support risk stratification rather than treating every VTS diagnosis as a uniformly high-risk state.

Large contemporary cohorts strengthen the timing signal. Among 38,876 ART singletons, demise after fetal cardiac activity was associated with approximately doubled risks of preterm birth (adjusted risk ratio 2.00, 95% CI 1.77–2.24) and perinatal mortality (adjusted risk ratio 2.19, 95% CI 1.12–4.30) and with increased low birthweight and small-for-gestational-age birth, whereas demise occurring before cardiac activity had been documented was associated with attenuated and largely non-significant increases in the same outcomes [44]. A prospective multicenter cohort of 4746 singleton pregnancies, in which VTS occurred in 1.1% (54 pregnancies), identified VTS as an independent risk factor for both spontaneous and medically indicated preterm birth, although the small number of exposed pregnancies limits precision [45]. In an IVF cohort of 636 survivors stratified into early (≤13 weeks, n = 593) and late (>13 weeks, n = 43) VTS, both categories had lower birthweight than primary singletons, but late VTS carried the larger penalty on a very small denominator [46]. The clinically useful hierarchy is therefore: ongoing twins generally carry the greatest global prematurity burden; VTS survivors occupy an intermediate position; and primary singletons have the lowest baseline risk.

### 5.3. Long-Term Outcomes in the Surviving Child

Long-term data remain the weakest part of the evidence base. The Danish cohort found no excess of neurological sequelae overall but a correlation between later onset of spontaneous reduction and neurological morbidity, together with the increased mortality noted above [20]. A recent propensity-matched follow-up of frozen-embryo-transfer singletons (66 exposed, 264 unexposed) found no difference in growth or developmental measures up to 3 years of age, although the sample was small and the horizon short [47]. Population data also indicate that the cerebral palsy risk associated with ART has declined as single-embryo transfer has been adopted [48], which is consistent with, but not proof of, a causal contribution from early multiple implantation. No study has yet reported cardiometabolic or school-age cognitive outcomes specifically after VTS.

Whether the sex of the surviving child is altered has been asked but not adequately answered. Infant sex is not among the outcomes pooled in the largest systematic review of VTS in ART singletons [42], and where individual cohorts have tabulated it, the results are inconsistent and are drawn from small numbers. The intuition that male conceptuses are preferentially lost, which underlies much of this discussion, is itself questionable: analysis of the human sex ratio from conception onward indicates that the ratio at conception is close to parity and that female mortality exceeds male mortality during much of gestation [49]. At the population level, the sex ratio of twins at term has been used as an indirect marker of vanishing-twin survivors in ecological time-series analyses of United States birth records, which have related it to birthweight in periviable singletons and to infant mortality [50,51]; these analyses concern predominantly spontaneous conceptions, infer rather than observe the exposure, and their authors note that vanishing usually precedes clinical recognition of pregnancy. Critically, no study has compared the sex of the survivor with the sex of the vanished co-twin, because the latter is almost never determined. Cell-free DNA offers a feasible route, since a Y-chromosome signal in a woman carrying a female survivor identifies a male co-twin, and such discrepancies are already encountered incidentally in screening programs; assembling them prospectively would answer the question directly.

## 6. Conception Mode as an Effect Modifier, and the Distinction from Iatrogenic Fetal Reduction

In two consecutive reports from a single tertiary center, Márton et al. compared VTS after IVF/ICSI with VTS after spontaneous conception rather than assuming that all VTS pregnancies share one risk profile. In the 2016 report, 306 VTS pregnancies were analyzed, including 78 after IVF/ICSI and 228 after spontaneous conception, each matched to primary singleton controls [12]. The lower proportional prevalence among IVF/ICSI twins suggested that embryo selection could reduce early embryonic attrition within established twin pregnancies. Yet pregnancy complications, in particular pregestational and gestational diabetes, were concentrated in the IVF/ICSI subgroup, and the authors concluded that perinatal outcome after VTS was worse following IVF/ICSI than after spontaneous conception. This apparently paradoxical combination—fewer reductions among twins but less favorable outcome when reduction occurs—argues against using prevalence as a proxy for severity.

The expanded 2020 cohort comprised 316 VTS pregnancies, 81 after IVF/ICSI and 235 after spontaneous conception, recruited between 1994 and 2016 [16]. Compared with spontaneous VTS, IVF/ICSI VTS was more strongly associated with pregestational diabetes and gestational diabetes (adjusted odds ratios 4.12 and 11.1, respectively) and with placental insertion abnormalities. Within IVF pregnancies, VTS was a major prognosticator of intrauterine growth restriction in the surviving fetus (adjusted odds ratio 5.12), while advanced age, primiparity, hypertensive disorders, gestational diabetes, and placentation anomalies were independently associated with the VTS phenotype. These data place the placenta and the maternal metabolic–vascular environment at the center of the IVF-specific risk model.

These cohorts should nevertheless be interpreted within their retrospective single-center context and their long recruitment periods, during which embryo culture, transfer policy, cryopreservation, and obstetric surveillance changed. Residual confounding by infertility diagnosis, embryo number, and treatment era cannot be excluded; the number of IVF/ICSI cases in each report was modest, and some of the published odds ratios are reported without an explicit reference category. Their principal contribution is therefore not a universal causal estimate but a clinically coherent hypothesis: ART-related VTS may identify a subgroup in whom metabolic disease and abnormal placentation amplify the growth consequences of an early co-twin loss. This hypothesis is supported by the placental pathology described in an independent fresh-IVF cohort [33] and deserves prospective validation with standardized ultrasound phenotyping.

A separate distinction, frequently blurred in the literature, is between spontaneous reduction and iatrogenic multifetal pregnancy reduction. In a cohort of 1796 IVF/ICSI singletons comprising 271 spontaneously reduced, 84 surgically reduced, and 1441 primary singleton pregnancies, preterm birth and low birthweight were significantly more frequent after surgical reduction, whereas gestational age and birthweight after spontaneous reduction were comparable to those of primary singletons [52]. A large frozen-embryo-transfer cohort likewise analyzed the two exposures separately and found that both were associated with adverse outcomes relative to primary singletons, with late events more problematic than early ones [53]. Pooling spontaneous and iatrogenic reduction therefore risks attributing procedure-related risk to the biology of VTS, and the two should always be reported separately.

The principal peer-reviewed cohorts, meta-analyses, and their interpretations are tabulated in Supplementary Table S1.

## 7. Fresh Versus Frozen Embryo Transfer

The older VTS literature is dominated by fresh transfer and cannot be uncritically extrapolated to current practice. Frozen embryo transfer (FET) differs in corpus-luteum status, endometrial preparation, hormone exposure, embryo selection, and gestational dating, and it also carries a lower risk of monozygotic twinning than fresh transfer [24]. In a 2025 cohort of 14,583 singleton live births after FET, comprising 1078 VTS and 13,505 non-VTS pregnancies, VTS was associated with preterm birth (risk ratio 1.53, 95% CI 1.23–1.91) and low birthweight (risk ratio 1.76, 95% CI 1.34–2.32), and these associations were reported to be stable both before and after propensity-score matching [21]. An analysis of 33,238 ongoing IVF-FET pregnancies reported higher risks of preterm birth and low birthweight after both early and late VTS than after primary singleton implantation, and late VTS was additionally associated with abnormal placentation [53]. Both groups nevertheless compared favorably with nonreduced twins for several outcomes.

These FET data show that VTS is not simply an artifact of supraphysiologic fresh-cycle stimulation. They do not prove that cryopreservation or a specific endometrial regimen causes the association, because residual confounding and selection into live-birth cohorts remain. Two recent placental studies also caution against assuming a simple hormonal mechanism: supraphysiologic peri-trigger estradiol was not associated with clinically increased placental abnormalities after fresh transfer [54], and programmed and natural-cycle FET produced comparable rates of anatomic, inflammatory, maturation, and vascular placental disorders [55]. Future studies should nevertheless separate natural-cycle from programmed FET, cleavage-stage from blastocyst transfer, and single- from double-embryo transfer; report preimplantation genetic testing; and model the infertility diagnosis. Without those variables, “IVF-ET” remains an exposure bundle rather than a single causal factor.

## 8. Prenatal Screening and Genetic Counseling

A demised co-twin can distort biochemical and cell-free DNA screening. First-trimester serum markers after VTS differ according to whether the second sac is empty or contains a demised embryo and according to the interval since demise [56,57,58]. In 528 dichorionic pregnancies with a vanishing twin, free β-human chorionic gonadotropin was not significantly different from that of matched singletons, whereas pregnancy-associated plasma protein-A (PAPP-A) was approximately 17% higher in both the empty-sac and the demised-embryo subgroups; the authors therefore recommended that first-trimester screening rely on maternal age, fetal nuchal translucency, and free β-hCG, either omitting PAPP-A or adjusting it for the interval between estimated demise and blood sampling [57]. Earlier ART data are consistent in that markers in pregnancies with an early recognized vanishing twin did not differ from those of other ART singletons, while risk assessment was less reliable when demise was first identified at the nuchal translucency scan [56]. The requisition and ultrasound report should therefore document the original sac number, presence of a yolk sac or fetal pole, prior cardiac activity, estimated date of demise, and chorionicity.

Residual trophoblastic DNA from the demised conceptus may persist in maternal plasma, creating a false-positive noninvasive prenatal test, apparent sex discordance, or an uninterpretable result. In the TRIDENT-2 study of 655 vanishing-twin pregnancies, an abnormal cell-free DNA result was far more common when a non-viable embryo had been seen (type II) than when the additional sac was empty (type I) (12.6% versus 1.7%); testing appeared suitable for detecting trisomy 21 in the surviving fetus, whereas none of the trisomy 18, trisomy 13, or additional findings could be confirmed cytogenetically, and no discordant-positive result occurred when sampling was performed after 15 weeks [14]. Smaller series in multiple and vanishing-twin pregnancies report comparable patterns of raised no-result and false-positive rates [59,60]. A positive result must not automatically be attributed to the surviving fetus. Genetic counseling should address the VTS-specific limitations and offer diagnostic testing when the result, ultrasound, or clinical context warrants certainty. Deferring or repeating cell-free DNA analysis until after 15 weeks appears to reduce discordance substantially, although the available samples are too small to exclude residual risk.

## 9. Clinical Management and Counseling

No intervention has been shown to reverse an established early VTS. We are also unaware of any guideline or consensus statement dedicated to VTS: having examined current guidance from ESHRE, ASRM/SART, ISUOG, FIGO, ACOG, SMFM, RCOG, and NICE, we found no document that defines diagnostic criteria, classifies the condition, or specifies surveillance or counseling of the survivor. Where VTS appears, it is confined to first-trimester aneuploidy screening: ISUOG and FIGO advise nuchal translucency with maternal age in place of serum biochemistry when a twin has vanished [8,61], ACOG and SMFM note it only as a cause of discordant or uninterpretable cell-free DNA results, and the ASRM committee opinion on multiple gestation describes VTS epidemiology without issuing a recommendation [62]; RCOG guidance on late intrauterine death explicitly excludes multiple pregnancies with a surviving fetus from its scope. Published appraisals of twin-pregnancy guidance reach the same conclusion [63]. The first task is accurate classification: confirm viability of the survivor, date the loss, establish chorionicity when possible, and exclude threats to the ongoing pregnancy. Uncomplicated disappearance of an empty dichorionic sac in the early first trimester often requires no treatment beyond routine obstetric care and clear safety-net instructions. Loss after fetal cardiac activity, later first-trimester loss, monochorionic placentation, persistent bleeding, or maternal comorbidity justifies a lower threshold for maternal–fetal medicine review; after single fetal death in a monochorionic pregnancy, fetal brain imaging in the survivor carries additional prognostic weight [15].

Surveillance should be proportional to the phenotype rather than driven by the label alone. A detailed anatomic examination and careful assessment of placental morphology and cord insertion are reasonable after IVF-ET VTS. Third-trimester growth assessment is particularly defensible when loss occurred after cardiac activity, when placental abnormalities are present, or when diabetes, hypertension, or prior fetal growth restriction adds risk [16,44]. No trial has evaluated aspirin, progesterone, anticoagulation, or intensified fetal testing in this population, and there is accordingly no evidence to support prescribing them solely because an early empty sac vanished; these interventions should follow their usual indications. Recommendations in this section are therefore consensus-based rather than trial-based, and should be presented to patients as such.

Communication is part of care. Patients may experience simultaneous grief for the demised twin and anxiety or relief regarding the survivor. In a global mixed-methods survey of 153 patients from 17 countries, mean satisfaction with the amount of information received was 3.5 out of 10, most respondents rated their interaction with clinicians negatively, and 43% were never informed about chorionicity [64]; a companion review found international guidance on VTS to be inconsistent and largely silent on bereavement care [63]. Clinicians should name the loss, explain what is known and uncertain, avoid implying that the event is trivial, and provide a written plan for screening and follow-up. Psychological support should be offered without treating grief as evidence that the ongoing pregnancy is unwanted or unappreciated.

## 10. Prevention in IVF-ET

The most effective preventive strategy is to avoid creating a dizygotic multiple implantation. Peer-reviewed ESHRE guidance concluded that no clinical or embryological factor per se justifies double rather than elective single-embryo transfer because cumulative live-birth probability can be preserved while multiple-pregnancy risk is reduced [65]. ASRM and SART likewise recommend age-, prognosis-, and embryo-stage-specific limits and favor single transfer for euploid embryos and favorable-prognosis blastocysts [66]. A Cochrane review of randomized trials confirmed that repeated single-embryo transfer achieves a cumulative live-birth rate close to that of double-embryo transfer while substantially reducing multiple pregnancy [67], and registry data from 133 German centers and more than 22,000 cycles showed that two successive single transfers yielded more women with a live birth than one double transfer (45.6% versus 38.4% per woman), with far fewer multiple births (1.7% versus 30.3% per live-birth delivery) and fewer preterm births (942/5196, 18.1%, versus 2619/5632, 46.5%, of liveborn children) [68]. The three proportions use different denominators and should not be read as a single series; the preterm excess in the double-transfer arm is driven predominantly by preterm multiples, who accounted for 36.7% of all children born in that arm compared with 2.7% after two successive single transfers, while preterm singletons accounted for 9.8% and 15.5%, respectively. This is not merely prevention of twin delivery: it prevents the intermediate VTS state and its diagnostic, placental, and psychological consequences [9].

Counseling before transfer should explicitly include VTS when more than one embryo is considered. Patients should understand that spontaneous reduction does not convert a multiple implantation into a biologically identical primary singleton, and that transfer of additional embryos trades a possible cycle-level gain against risks to both maternal and offspring health. Single-embryo transfer cannot prevent monozygotic splitting, particularly after blastocyst transfer, but the residual risk is small compared with the avoidable risk created by transferring multiple embryos [22,24,65,66]. It should also be acknowledged that dichorionic twin pregnancies arising after single-embryo transfer are predominantly monozygotic and may behave less favorably than dizygotic dichorionic twins after double transfer [69], which is a further argument for counseling that addresses zygosity rather than embryo number alone.

## 11. Research Priorities

Future studies should adopt a core VTS dataset, summarized in Table 1: conception mode; infertility diagnosis; fresh or frozen transfer and endometrial regimen; embryo number, stage, morphology, and genetic testing; initial sac and embryo number; type I versus type II loss; chorionicity; cardiac activity; exact gestational age at loss; placental pathology; and standardized maternal and neonatal outcomes. Analyses should use primary ART singletons and ongoing twins as separate comparators, report spontaneous and iatrogenic reduction separately, avoid conditioning only on live birth when studying perinatal mortality, and report absolute risks alongside adjusted relative measures. Prospective multicenter cohorts could test whether the metabolic and placental pathway suggested by the conception-mode comparisons identifies a reproducible high-risk subgroup [12,16]. Long-term neurodevelopmental and cardiometabolic follow-up remains especially sparse: the only dedicated follow-up study extends to 3 years in 66 exposed children [47], and no cohort has yet linked first-trimester VTS phenotype to school-age outcomes.

## 12. Strengths and Limitations of This Review

This review is deliberately restricted to peer-reviewed evidence, prioritizes studies with an ART-conceived comparator and explicit timing of loss, and presents discordant findings rather than a single pooled estimate. Its limitations follow from the same design. The search was narrative rather than systematic, was limited to English-language reports, and did not include formal risk-of-bias appraisal or a registered protocol; selection of studies therefore involved judgement and some relevant reports may have been missed. Almost all available evidence is retrospective and observational, several of the pivotal cohorts are single-center, and overlapping registry populations may contribute more than once to the pooled estimates cited here. Because definitions of VTS, first-scan timing, and outcome ascertainment differ between studies, the effect sizes quoted are not directly comparable, and no quantitative synthesis was attempted. The recommendations in Section 9, Section 10 and Section 11 are consensus-based extrapolations from observational data and are not supported by interventional evidence.

## 13. Conclusions

VTS after IVF-ET is not a single prognostic entity. The strongest determinants are developmental stage at demise, gestational timing, chorionicity, placental phenotype, and maternal metabolic–vascular risk. The surviving singleton generally fares better than an ongoing twin, but may retain excess risk relative to a primary singleton, especially after loss of an embryo with cardiac activity or later demise, whereas cohorts restricted to viable deliveries and the earliest pooled analysis found no measurable penalty. Direct comparisons of conception mode, presently limited to two overlapping single-center cohorts, suggest that VTS may be proportionally less frequent among IVF/ICSI twins yet more clinically consequential when it occurs, with fetal growth restriction and abnormal placentation as central signals; independent replication is required before this is treated as established knowledge. Accurate early ultrasound phenotyping using an agreed classification, VTS-aware aneuploidy screening, individualized placental and growth surveillance, sensitive communication, and elective single-embryo transfer provide the most defensible current clinical framework, while adequately powered prospective cohorts with long-term follow-up remain the principal unmet research need.

## Figures and Tables

**Table 1:** Proposed minimum reporting set for studies of vanishing twin syndrome after IVF-ET.

Domain	Items That Should Be Reported
Conception and treatment	Conception mode; infertility diagnosis; fresh or frozen transfer; endometrial preparation (natural cycle versus programmed); embryo stage and morphological grade; number of embryos transferred; assisted hatching; preimplantation genetic testing status
Initial ultrasound phenotype	Gestational age at first scan; number of gestational sacs; presence of yolk sac and fetal pole in each sac; documented cardiac activity per sac; chorionicity and amnionicity
Characteristics of the loss	Type I (additional empty gestational sac) versus type II (additional non-viable embryo); exact gestational age at demise; presence and volume of intrauterine or subchorionic hematoma; interval between demise and any biochemical or cell-free DNA sampling
Comparators	Primary ART singletons and ongoing (nonreduced) twins analyzed as separate comparator groups; spontaneous reduction reported separately from iatrogenic multifetal pregnancy reduction
Outcomes	Gestational age; birthweight; small-for-gestational-age status with the reference chart specified; preterm birth by subtype (spontaneous versus medically indicated); perinatal mortality not conditioned on live birth; placental weight and pathology using a standard nosology; maternal metabolic and hypertensive disease
Follow-up	Neonatal morbidity; childhood growth; neurodevelopment; cardiometabolic outcomes

Abbreviations: ART, assisted reproductive technology; IVF-ET, in vitro fertilization and embryo transfer.

## Data Availability

No new data were created or analyzed in this study. Data sharing is not applicable to this article.

## Supplementary Materials

The following supporting information can be downloaded at https://www.mdpi.com/article/10.3390/biomedicines14092036/s1. Table S1: Selected peer-reviewed evidence on vanishing twin syndrome after IVF-ET.
