# Supplementary material for: Vanishing Twin Syndrome After In Vitro Fertilization and Embryo Transfer: Mechanisms, Perinatal Consequences, Diagnostic Pitfalls, and Prevention
Source: Biomedicines. 2026 Sep 10;14(9):2036. doi: 10.3390/biomedicines14092036 (PMC13604789; doi:10.3390/biomedicines14092036)
Supplement: Supplementary file 1 [file biomedicines-14-02036-s001.zip › biomedicines-4556392-supplementary.pdf]

Supplementary Material

Vanishing Twin Syndrome After In Vitro Fertilization and Embryo Transfer: Mechanisms, Perinatal Consequences, Diagnostic Pitfalls, and Prevention

This table was presented as Table 1 in the originally submitted manuscript and has been moved to the Supplementary Material in response to reviewer comment. Reference numbers correspond to the reference list of the main manuscript.

Table S1. Selected peer-reviewed evidence on vanishing twin syndrome after IVF-ET.

Study	Design and population	Principal IVF-ET finding	Interpretation
Márton et al., 2016 [12]	Retrospective cohort; 306 VTS pregnancies (78 IVF/ICSI, 228 spontaneous) over 22 years, matched to primary singletons	VTS in 12.6% of IVF/ICSI versus 18.2% of spontaneous twin pregnancies; complications, notably diabetes, concentrated in the IVF/ICSI subgroup	Prevalence and severity should not be conflated
Márton et al., 2020 [16]	Retrospective cohort; 316 VTS pregnancies (81 IVF/ICSI, 235 spontaneous), 1994–2016	Pregestational diabetes and gestational diabetes more strongly associated with VTS after IVF/ICSI (aOR 4.12 and 11.1); placental insertion abnormalities more frequent; VTS predicted IUGR within IVF pregnancies (aOR 5.12)	Supports a metabolic–placental risk pathway
Sun et al., 2017 [41]	Systematic review and meta-analysis; 5 observational studies of ART VTS versus singleton-at-start	No difference in gestational age or overall preterm delivery; extremely preterm delivery increased (RR 3.5, 95% CI 1.72–7.12); mean birthweight marginally lower	Early pooled evidence was underpowered and unstable
Li et al., 2020 [42]	Systematic review and meta-analysis; 17 studies, >60,000 ART singletons	Outcome depended on timing of loss and growth stage reached; no gestational-age penalty for loss at or before 14 weeks	Empty sac and later fetal demise should be analyzed separately
Zhou et al., 2020 [43]	Systematic review and meta-analysis; 15 studies	Preterm birth <37 weeks OR 2.41 (1.46–3.99) and SGA OR 2.70 (1.39–5.24) versus ART singletons; malformations not increased (OR 1.25, 0.87–1.79)	Moderate excess perinatal risk without excess malformation
Harris et al., 2020 [33]	Retrospective cohort; 905 fresh autologous IVF cycles with placental pathology, including 73 VTS	No excess preterm birth or growth restriction versus IVF singletons; more small placentas and anatomic placental abnormalities (aOR 2.15, 1.08–4.28)	Placental phenotype may change without a detectable perinatal signal

Study	Design and population	Principal IVF-ET finding	Interpretation
Zhu et al., 2020 [44]	Retrospective cohort; 38,876 ART singleton births	Loss after cardiac activity: preterm birth aRR 2.00 (1.77–2.24), perinatal mortality aRR 2.19 (1.12–4.30); loss before cardiac activity attenuated	Developmental stage at loss is prognostically important
He et al., 2025 [21]	Retrospective FET cohort; 14,583 singleton live births, 1078 VTS	VTS associated with preterm birth (RR 1.53) and low birthweight (RR 1.76) after propensity-score matching	Risk persists in contemporary FET practice
Zhang et al., 2026 [53]	Retrospective IVF-FET cohort; 33,238 ongoing pregnancies, 1354 VTS, 116 fetal reductions	Early and late VTS increased preterm birth and low birthweight versus primary singletons; late VTS increased abnormal placentation; outcomes similar to or better than nonreduced twins	Late loss carries the greatest placental and perinatal signal
Jing et al., 2026 [52]	Retrospective cohort; 1796 IVF/ICSI singletons (271 spontaneous reduction, 84 surgical reduction, 1441 primary singletons)	Gestational age and birthweight after spontaneous reduction comparable to primary singletons; preterm birth and low birthweight increased after surgical reduction	Discordant evidence; spontaneous and iatrogenic reduction must be separated
Yu et al., 2026 [47]	Retrospective propensity-matched cohort; 66 VTS and 264 non-VTS FET singletons followed to 3 years	No difference in growth or developmental measures up to 3 years	Reassuring but small; long-term evidence remains scarce

Abbreviations: aOR, adjusted odds ratio; aRR, adjusted risk ratio; ART, assisted reproductive technology; CI, confidence interval; FET, frozen embryo transfer; ICSI, intracytoplasmic sperm injection; IUGR, intrauterine growth restriction; IVF-ET, in vitro fertilization and embryo transfer; OR, odds ratio; RR, risk ratio; SGA, small for gestational age; VTS, vanishing twin syndrome.
